# The comorbidity and cognition in multiple sclerosis (CCOMS) neuroimaging protocol: Study rationale, MRI acquisition, and minimal image processing pipelines

**DOI:** 10.3389/fnimg.2022.970385

**Published:** 2022-08-24

**Authors:** Md Nasir Uddin, Teresa D. Figley, Jennifer Kornelsen, Erin L. Mazerolle, Carl A. Helmick, Christopher B. O'Grady, Salina Pirzada, Ronak Patel, Sean Carter, Kaihim Wong, Marco R. Essig, Lesley A. Graff, James M. Bolton, James J. Marriott, Charles N. Bernstein, John D. Fisk, Ruth Ann Marrie, Chase R. Figley

**Affiliations:** ^1^Department of Radiology, Max Rady College of Medicine, Rady Faculty of Health Sciences, University of Manitoba, Winnipeg, MB, Canada; ^2^Division of Diagnostic Imaging, Health Sciences Centre Winnipeg, Winnipeg, MB, Canada; ^3^Neuroscience Research Program, Kleysen Institute for Advanced Medicine, Health Sciences Centre Winnipeg, Winnipeg, MB, Canada; ^4^Department of Physiology and Pathophysiology, Max Rady College of Medicine, Rady Faculty of Health Sciences, University of Manitoba, Winnipeg, MB, Canada; ^5^Department of Psychology, St. Francis Xavier University, Antigonish, NS, Canada; ^6^Division of Geriatric Medicine, Department of Psychiatry, Dalhousie University, Halifax, NS, Canada; ^7^Department of Anesthesia and Biomedical Translational Imaging Centre, Dalhousie University, Halifax, NS, Canada; ^8^Department of Clinical Health Psychology, Max Rady College of Medicine, Rady Faculty of Health Sciences, University of Manitoba, Winnipeg, MB, Canada; ^9^Department of Psychiatry, Max Rady College of Medicine, Rady Faculty of Health Sciences, University of Manitoba, Winnipeg, MB, Canada; ^10^Department of Internal Medicine, Max Rady College of Medicine, Rady Faculty of Health Sciences, University of Manitoba, Winnipeg, MB, Canada; ^11^Nova Scotia Health Authority and the Departments of Psychiatry, Psychology and Neuroscience, and Medicine, Dalhousie University, Halifax, NS, Canada; ^12^Department of Community Health Sciences, Max Rady College of Medicine, Rady Faculty of Health Sciences, University of Manitoba, Winnipeg, MB, Canada

**Keywords:** brain, cognition, comorbidities, MRI, multiple sclerosis, neuroimaging

## Abstract

The Comorbidity and Cognition in Multiple Sclerosis (CCOMS) study represents a coordinated effort by a team of clinicians, neuropsychologists, and neuroimaging experts to investigate the neural basis of cognitive changes and their association with comorbidities among persons with multiple sclerosis (MS). The objectives are to determine the relationships among psychiatric (e.g., depression or anxiety) and vascular (e.g., diabetes, hypertension, etc.) comorbidities, cognitive performance, and MRI measures of brain structure and function, including changes over time. Because neuroimaging forms the basis for several investigations of specific neural correlates that will be reported in future publications, the goal of the current manuscript is to briefly review the CCOMS study design and baseline characteristics for participants enrolled in the three study cohorts (MS, psychiatric control, and healthy control), and provide a detailed description of the MRI hardware, neuroimaging acquisition parameters, and image processing pipelines for the volumetric, microstructural, functional, and perfusion MRI data.

## Introduction

Although there are a variety of disease-modifying therapies (Rae-Grant et al., 2018; Gholamzad et al., 2019) available to reduce relapses and slow disability progression in multiple sclerosis (MS), the efficacy of these treatments is limited and there is still no cure for this disease. In addition to the underlying neuroinflammatory and neurodegenerative disease mechanisms associated with MS, physical comorbidities such as diabetes and hyperlipidemia accelerate the overall rate of disability progression in MS (Zhang et al., 2018) and increase the risk of relapse in relapsing-remitting MS (Kowalec et al., 2017). Similarly, elevated cardiovascular risk among persons with MS is associated with higher relapse rate, increased disability, and escalation of disease modifying therapies in MS (Petruzzo et al., 2021), and with poorer cognitive performance (Reia et al., 2021). Furthermore, common psychiatric comorbidities including anxiety disorders and depression decrease quality of life and cognitive performance, and are associated with faster progression of disability in MS (McKay et al., 2018; Marrie et al., 2019; Palladino et al., 2021). Therefore, these physical and psychiatric comorbidities represent potentially modifiable factors that could be targeted to improve patient outcomes.

However, relatively little is known about the relationships between these common comorbidities and cross-sectional or longitudinal differences in brain anatomy and physiology. The Comorbidity and Cognition in Multiple Sclerosis (CCOMS) study was designed to examine these issues, with a focus on cognitive impairment and decline, which affects between 40 and 65% of individuals with MS (Rao et al., 1991a; Rao, 1995) and has profound impact on their psychological functioning, ability to maintain employment, and overall wellbeing (Rao et al., 1991b; Morrow et al., 2010).

The CCOMS study was originally designed as a 2-year longitudinal neurologic, psychiatric, cognitive, and radiologic evaluation of a cohort of over 100 persons with MS. These individuals were recruited from an ongoing longitudinal comparative study of three immune-mediated inflammatory diseases (the “IMID” study) (Marrie et al., 2017, 2018a; Whitehouse et al., 2019). The overarching goal of the CCOMS sub-study was to evaluate how depression, anxiety, diabetes and hypertension affect cognitive function in MS (Marrie et al., 2019), and to examine how brain structure and function are related to these factors. As the IMID study did not include a neuroimaging component, we sought to develop and implement a comprehensive brain MRI protocol to examine different aspects of brain macrostructure (e.g., regional volumes and cortical thickness), microstructure (e.g., tissue density, myelin concentration, and iron accumulation), and function (e.g., resting state blood-oxygen fluctuations and cerebral perfusion). Subsequently, with the availability of additional funding, cross-sectional evaluations of a cohort of over 50 “psychiatric” control participants (with depression and/or anxiety) in the IMID study, and a cohort of 100 “healthy” control participants (with no physical or psychiatric comorbidities) were also performed using the same MRI protocol.

The neuroimaging protocol for the CCOMS study, which includes several advanced structural, functional, and perfusion MRI measures, was not part of the original IMID study and has not been previously reported in full (Pirzada et al., 2020; Marrie et al., 2021a, 2022). Thus, the goal of the current paper is to provide a detailed overview of the neuroimaging acquisition parameters and pre-processing methods that have been employed to serve as a technical reference for subsequent study papers. In particular, the remainder of this paper provides: (1) a brief description of the MS and control participants; (2) a comprehensive outline of the MRI hardware, pulse sequences, and parameters; (3) a thorough overview of the image processing pipelines for each data type, including anatomical imaging, diffusion imaging, myelin water imaging, resting state fMRI, perfusion imaging, MR relaxometry, and quantitative susceptibility mapping; and (4) a discussion of the strengths and weaknesses of our approach.

## Overview of study participants

The IMID study recruited cohorts of individuals with MS, inflammatory bowel disease, rheumatoid arthritis, or a lifetime history of a psychiatric disorder who did not have an immune-mediated inflammatory disease, to examine commonalities and differences across IMIDs regarding the impact of psychiatric disorders on several outcomes (Marrie et al., 2018a). For the CCOMS sub-study, a subgroup of participants with MS from the IMID study was recruited to undergo neuroimaging and additional cognitive and psychiatric testing. Following the procurement of additional funding, a cohort of psychiatric controls (also drawn from the parent IMID study) and a cohort of healthy controls were recruited. The University of Manitoba Health Research Ethics Board approved the IMID study, and related sub-studies. All participants were ≥18 years of age, had sufficient knowledge of English to complete study questionnaires, and provided written informed consent before enrollment.

After verbal screening to exclude participants with contraindications for MRI, and any history of brain tumor, neurodegenerative disorder, and/or immune-mediated inflammatory disease (except for MS in the case of participants being recruited into that study cohort), 111 people with MS, 61 psychiatric controls with a lifetime history of depression and/or anxiety, and 108 healthy controls initially agreed to participate. Ultimately, baseline MRI data were acquired from 111 participants in the MS cohort, 52 participants in the psychiatric control cohort, and 100 participants in the healthy control cohort.

### Multiple sclerosis (MS) cohort

All 111 participants in the MS cohort were recruited from the IMID study (Marrie et al., 2018a), which employed medical records review to confirm dates of MS diagnoses based on prevailing criteria at the time (McDonald et al., 2001; Polman et al., 2005, 2011; Thompson et al., 2018). Disease activity was assessed using the annualized relapse rate, and disease severity was assessed using the Expanded Disability Status Scale (EDSS) (Kurtzke, 1983) at each study visit. Disease-modifying therapies used, and categories of concomitant medications were captured according to the World Health Organization's Anatomical Therapeutic Chemical Classification (WHO-ATC) System at each visit. Since a major aim of the CCOMS study was to investigate psychiatric comorbidities in MS, participants with depression and/or anxiety were not excluded.

### Psychiatric control cohort

All 52 participants in the psychiatric cohort were also recruited from the IMID study (Marrie et al., 2018a). As described previously, participants in the psychiatric cohort were recruited from Winnipeg-based primary and tertiary mental health care settings, via posters and word of mouth. Diagnoses of depression or anxiety were confirmed at the first study visit using the structured clinical interview for DSM-IV-TR Axis I Disorders (SCID)—Research Version (First et al., 2002). Current medications used were captured according to the WHO-ATC system.

### Healthy control (HC) cohort

As described previously, healthy control participants were recruited using posters placed in multiple settings throughout Winnipeg, direct mail to homes in the city, and word of mouth (Marrie et al., 2021b). Exclusion criteria specific to this cohort included any history of traumatic brain injury associated with loss of consciousness or amnesia; any chronic medical condition (including self-reported hypertension or blood pressure > 140/90 identified during the study visit); known intellectual disability or cognitive impairment; self-reported psychiatric condition or positive response to the SCID-IV screening questions for depressive or anxiety disorders; and chronic medication use other than contraceptives or hormone replacement therapy.

### Brief demographic comparison of MS vs. control cohorts

Characteristics of the three CCOMS study cohorts, as well as a breakdown by disease subtype (i.e., RRMS, SPMS, PPMS) in the MS cohort, are summarized in Table 1. Participants in all three groups were mostly middle-aged and predominantly female. However, while the psychiatric cohort was not statistically different compared to the MS cohort based on sex (*p* = 0.49; two-tailed difference of proportions test), age, education level, or disease duration (all *p* ≥ 0.05; two-tailed *t*-tests), the HC cohort had a lower proportion of females than the MS cohort (two-tailed *p* < 0.005), was younger, and had a higher level of education than the MS cohort (both two-tailed *p* < 0.001).

**Table 1:** Baseline demographic and clinical data for participants in each study cohort.

**Variables**	**Multiple sclerosis cohort [at first MRI]**				**Healthy control cohort**	**Psychiatric cohort**	***P*-values** **(MS ≠ HC** **MS ≠ NP)**
	**All**	**RRMS**	**SPMS**	**PPMS**			
Number of participants	111	93	12	6	100	52	N/A
Gender (male/female)	19/92	17/76	2/10	0/6	35/65	12/40	***p*** **<** **0.005** *p* = 0.49
Age (years)^a^ [mean ±*SD*]	49.59 ± 12.71	47.26 ± 12.15	61.11 ± 8.09	62.60 ± 8.54	39.05 ± 16.24	48.63 ± 12.64	***p*** **<** **0.001** *p* = 0.65
Years of education [mean ±*SD*]	13.99 ± 2.55	14.18 ± 2.51	12.42 ± 2.64	14.17 ± 2.23	16.76 ± 2.98	14.88 ± 2.88	***p*** **<** **0.001** p = 0.05
Disease type^b^	93/12/6	93	12	6	N/A	19/6/27	N/A N/A
Disease duration (years) ^c^ [mean ±*SD*]	20.25 ± 12.16	18.81 ± 12.16	27.10 ± 11.73	18.66 ± 16.22	N/A	N/A	N/A N/A
Baseline EDSS score ^d^ [median (IQR)]	3.50 (2.5–4.0)	3.16 (2.5–4.0)	5.04 (4.38–6.5)	4.14 (4.0–6.0)	N/A	N/A	N/A N/A

Categorical values are reported as the number of participants and all other values are reported as mean ± standard deviation. For the MS Cohort, values are reflective of the first (baseline) MRI visit. EDSS, Expanded Disability Status Scale; IQR, Interquartile Range; N/A, Not Applicable.

^a^Age Range = 21–80 years; ^b^RRMS/SPMS/PPMS (MS) and Depression Only/Anxiety Only/Both Depression and Anxiety (NP) ^c^Disease Duration Range: 2–52 years (for All MS); ^d^Mean ± SD EDSS = 3.50 ± 1.49 (Total Range = 0–6.5).

Note: All p-values were calculated using two-tailed t-tests, except for gender (which was based on a two-tailed test for equality of proportions with continuity correction, “prop.test” in the R software package). Statistically significant p-values are indicated in bold. Some characteristics of the MS cohort (Marrie et al., 2019), and 103 healthy controls (Marrie et al., 2021b)—including three additional participants who were removed from the MRI dataset due to incidental findings—have been previously reported.

## Non-MRI data acquisition

### Sociodemographic information and clinical characteristics

Sociodemographic and behavioral characteristics were obtained for all participants at baseline, following the IMID study protocol (Marrie et al., 2018a). These included gender, date of birth, race and ethnicity, highest level of education attained and years of education, annual household income, marital status, having children, occupation, and smoking status. Participants reported their comorbidities, including diabetes, hypertension, and hyperlipidemia using a validated questionnaire (Horton et al., 2010), and indicated whether they were currently treated. Participants also underwent assessments of height and weight for the purposes of calculating body mass index. Blood pressure was measured while seated using an automated blood pressure machine, and serum HbA1c was measured using a blood sample (see Blood sample collection and storage below) at the time of the study visit. As noted previously, medication use was captured according to WHO-ATC class. Finally, lower limb function was measured using the Timed 25-Foot Walk (Motl et al., 2017), and upper limb function was measured using the Nine-Hole Peg Test (Feys et al., 2017).

### Cognitive assessments

For the CCOMS study, participants in the MS cohort also completed cognitive and psychiatric morbidity assessments at two time points, separated by 2 years. The psychiatric and healthy control participants completed these assessments at a single time point. To measure cognitive performance, all participants completed an expanded version of the IMID study neuropsychological assessment (Marrie et al., 2018a). This included all components of the Brief International Cognitive Assessment for Multiple Sclerosis (BICAMS) (Langdon et al., 2012) and most components of the Minimal Assessment of Cognitive Function in MS (MACFIMS) (Benedict et al., 2002), including: information processing speed, verbal learning and memory, visual learning and memory, and verbal fluency/executive ability. The Symbol Digit Modalities Test (Smith, 1973) measured processing speed, the Wechsler Memory Scale-III Letter-Number Sequencing sub-test measured working memory (Wechsler, 2008), the California Verbal Learning Test II measured verbal learning and memory (Delis et al., 2000), and the Brief Visuospatial Memory Test-Revised (BVMT-R) (Benedict and Hopkins, 2001) measured visual learning and memory. Language and executive abilities were measured using tests of verbal fluency (letter and animal categories) (Strauss et al., 2006) as well as the Delis-Kaplan Executive Function System (D-KEFS) Color-Word Interference Test (Delis, 2001). The Wechsler Test of Adult Reading (Wechsler, 2001) was also included as an estimate of premorbid intelligence.

### Psychiatric assessments

To evaluate psychiatric comorbidity, participants in all cohorts completed the Patient Health Questionnaire (PHQ-9) (Kroenke et al., 2003), Hospital Anxiety and Depression Scale (HADS) (Snaith, 2003), Kessler-6 Distress Scale (Kessler et al., 2003), PROMIS Emotional Distress Depression Short-Form 8a (PROMIS Depression) and Anxiety Short-Form 8a (PROMIS Anxiety) (Pilkonis et al., 2011), Generalized Anxiety Disorder 7-item Scale (GAD-7) (Spitzer et al., 2006), the Overall Anxiety and Severity Impairment Scale (OASIS) (Norman et al., 2006), and completed a structured clinical interview for DSM-IV-TR Axis I Disorders (SCID-IV) (First et al., 2002), as described previously (Marrie et al., 2018a,b). All participants in the MS and psychiatric cohorts, who were recruited through the IMID study, were administered an updated SCID for depression and anxiety disorders, which allowed us to confirm continuing diagnoses and identify any new diagnoses that may have emerged since enrollment in the IMID study.

### Blood sample collection and storage

As reported for the larger IMID study (Marrie et al., 2018a), consenting participants in all cohorts provided blood samples in EDTA and Paxgene (Qiagen) DNA sample tubes. After centrifuging, the plasma layer from the EDTA sample tubes was placed in vials for storage at −80°C; and DNA sample tubes were processed according to manufacturer instructions.

## MRI data acquisition

Participants in the MS cohort underwent two MRI scanning sessions 2 years apart, each within 4 weeks of the corresponding cognitive and psychiatric assessments. Participants in the psychiatric and HC cohorts completed a single MRI scanning session, within 4 weeks of the cognitive and psychiatric study visit. For all participants, T_1_-weighted and T_2_-weighted fluid attenuated inversion recovery MRI scans (details below) were reviewed by an experienced neuroradiologist (MRE) to screen for clinically relevant incidental findings.

All brain imaging data were acquired using the same 3T Siemens TIM Trio MRI system (software version VB17a) equipped with a Siemens TQ-Engine gradient set (maximum gradient strengths of 40 mT/m in the X and Y directions and 45 mT/m in the Z direction, with a slew rate of 200 T/m/s) and a Siemens 32-channel receive-only head coil (Siemens Healthcare, Erlangen, Germany). Each MRI session included: (1) T_1_-weighted, (2) dual-echo PD-weighted and T_2_-weighted, (3) T_2_-weighted with fluid attenuated inversion recovery (T_2_-FLAIR), (4) high angular resolution diffusion imaging (HARDI), (5) resting state fMRI (rs-fMRI), and (6) pseudo-continuous arterial spin labeling (pCASL) perfusion scans. Additional multi-component T_2_-relaxation myelin water imaging (MWI) and multi-echo gradient echo (MEGE) scans were acquired from participants in the MS cohort if they were able to tolerate a full 60-min MRI protocol, but these sequences were deemed to be a lower priority. Since participants in the control cohorts were unaccustomed to MRI, overall scan times were limited to 45-min and neither MWI nor MEGE data were acquired for these participants.

### Initial pilot testing and pulse sequence acquisition order

Since this was the first study at our site to implement multi-band pulse sequences (i.e., for HARDI, rs-fMRI, and pCASL), initial pilot testing was performed using a separate group of healthy volunteers who were not enrolled in the CCOMS study to establish the pulse sequence parameters that could be achieved on our 3T Siemens TIM Trio system within a single 1-h MRI session.

From this, we determined that a combination of hardware (primarily gradient strength) and overall scan-time limitations precluded the acquisition of multi-shell diffusion imaging data, which would have increased the echo-time (i.e., further decreasing image signal-to-noise ratio) and overall scan time, which would have negatively impacted our ability to include other pulse sequences of interest in the MRI protocol. We also experimented using different multi-band factors for the rs-fMRI sequence, since higher values are beneficial for increasing the sampling rate and improving statistical power, but can also cause more severe image distortions and spatially heterogeneous noise amplification (Risk et al., 2021). We ultimately chose to use a multi-band factor of 8, which is consistent with the Human Connectome Project (HCP) fMRI protocol (Van Essen et al., 2012; Smith et al., 2013) and has been empirically demonstrated to be within acceptable limits for studies done at 3T (or higher), using a 32-channel (or higher) head coil, and avoiding additional in-plane SENSE or GRAPPA accelerations (Xu et al., 2013). We complied with these caveats, visually inspected the pilot data (e.g., for excessive distortions), and included distortion correction (i.e., HySCO; described later) in the rs-fMRI preprocessing pipeline to mitigate distortion-related effects.

Finally, given the comparatively limited computational power of the Trio MRI console (single quad-core 2.67 GHz Intel^®^ Xeon^®^ CPU with 6 GB RAM and a single NVIDIA^®^ Quadro^®^ FX 3800 GPUs with 1 GB VRAM), pilot testing revealed long image reconstruction backlogs when trying to perform sequential acquisitions of large 4D images with the multi-band pulse sequences (i.e., HARDI, rs-fMRI, and pCASL). Therefore, the image acquisition order was optimized so that multi-band sequences were interleaved with more conventional and less reconstruction-intensive pulse sequences (i.e., T_2_-FLAIR, dual-echo PD/T_2_w turbo spin-echo, and fMRI field maps) so that the multi-band images could be fully reconstructed without exceeding the maximum image buffer permitted by the MRI console. Thus, the pulse sequences were acquired in the following order: three-plane localizer, T_1_w MPRAGE, anterior-posterior (blip-up) multi-band diffusion (HARDI), fMRI right-left field map, fMRI left-right field map, right-left multi-band rs-fMRI, axial T_2_-FLAIR, dual-echo PD/T_2_w turbo spin-echo, posterior-anterior (blip-down) multi-band diffusion (HARDI), multi-echo GRASE for myelin water imaging (MWI), multi-band perfusion (pCASL), MEGE for quantitative susceptibility mapping (QSM) and R${}_{2}^{*}$ (i.e., 1/T${}_{2}^{*}$) mapping. The main acquisition parameters for each pulse sequence are summarized in Table 2, and these and other details for each pulse sequence are provided below.

**Table 2:** Main acquisition parameters for each MRI sequence.

**Image type**	**Pulse sequence**	**Voxel size (mm)**	**Scan plane**	**Scan mode**	**FOV (mm) [freq. × phase]**	**Num slices**	**TR (ms)**	**TE (ms)**	**Excite/Refocus angle (deg)**	**Band-width (Hz/px)**	**Acceleration type and amount**	**Time**
T_1_w	MPRAGE	0.98/0.98/1.00	Ax/Sag	3D	250 **×** 250	176	1,900	2.47	9	170	GRAPPA 2	4:26
T_2_-FLAIR	FLAIR TSE	0.94/0.94/4.00	Ax	2D	240 **×** 240	32	9,000	100	90/130	287	N/A	5:06
PD/T_2_w	Dual Echo TSE	0.98/0.98/3.00	Ax	2D	250 **×** 187.5	54	4,500	11/101	90/150	250	N/A	3:45
HARDI	SE MB-EPI	2.00/2.00/2.00	Ax	2D	212 **×** 204	80	3,284	89.4	90/177	1,814	MB 4	3:17 (**×**2)
Fieldmap	SE MB-EPI	2.00/2.00/2.00	Ax	2D	208 **×** 180	72	10,170	86.6	90/180	2,290	MB 1	0:41 (**×**2)
rs-fMRI	GE MB-EPI	2.20/2.20/2.20	Ax	2D	220 **×** 171.6	80	1,000	38.6	61	2,272	MB 8	7:12
MWI	mGRASE	1.75/1.75/5.00	Ax	3D	224 **×** 168	22	1,500	10:10:320	90/180	1,260	N/A	16:53
pCASL	pCASL MB-EPI	3.44/3.44/4.50	Ax	2D	220 **×** 220	30	3,500	17	90	2,790	MB 6	4:47
R${}_{2}^{*}$/QSM	MEGE	1.33/1.33/1.33	Ax	3D	256 **×** 192	96	53	6:10:46	10	190	N/A	9:34

Additional details (e.g., matrix dimensions, inversion time, phase oversampling, refocusing flip angle, echo spacing, partial Fourier ratio, number of diffusion-encoding directions, and b-values, number of volumes, etc.) are provided in the text describing each sequence if/when appropriate. Some of the parameters for the T_1_w, T_2_-FLAIR, and PD/T_2_w sequences have been previously reported (Marrie et al., 2021a). Ax, Axial; EPI, Echo-Planar Imaging; FOV, Field Of View; GE, Gradient Echo; MB, Multiband; MEGE, Multi-Echo Gradient Echo; mGRASE, multi-echo gradient and spin echo; Sag, Sagittal; SE, Spin Echo; TSE, Turbo Spin Echo.

### T_1_-weighted anatomical scans

Whole-brain T_1_-weighted (T_1_w) images were acquired using a 3D magnetization prepared rapid acquisition gradient-echo (MPRAGE) sequence with the following scan parameters: repetition time [TR] = 1,900 ms, echo time [TE] = 2.47 ms, inversion time [TI] = 900 ms, flip angle = 9°, GRAPPA = 2, matrix size = 256 × 256, field of view [FOV] = 250 × 250 mm^2^, number of slices = 176, slice thickness = 1.00 mm, number of averages = 1, bandwidth [BW] = 170 Hz/Px, echo spacing [ESP] = 7.3 ms, spatial resolution = 0.98 × 0.98 × 1.00 mm^3^, acquisition time [TA] = 4:26 min.

The T_1_w MPRAGE data for participants in the MS cohort enrolled at the beginning of the study were acquired axially, but *post hoc* quality assurance evaluations revealed that images from a sub-set of participants contained relatively minor image artifacts. We therefore switched one of the 3D phase-encoding directions to acquire sagittal images, which eliminated the artifact in all subsequent study participants, including all controls and all of the 2-year follow-up scans for the MS cohort. We collected axial and sagittal oriented images from 32 controls to enable more detailed comparisons of the resulting brain volume estimates, which were in good agreement (see Supplementary material).

### Proton density and T_2_-weighted scans

Dual-echo proton density/T_2_-weighted (PD/T_2_w) images were obtained using a 2D turbo spin-echo sequence with the following scan parameters: TR = 4,500 ms, TE1/TE2 (PD/T_2_w) = 11/101 ms, flip angle = 90°, refocusing angle = 150°, matrix size = 256 × 192, FOV = 250 × 187.5 mm^2^, number of slices = 54; slice thickness = 3.00 mm, BW= 250 Hz/Px, turbo factor = 6, number of averages = 1, spatial resolution = 0.98 × 0.98 × 3.00 mm^3^, TA = 3:45 min.

### T_2_-weighted fluid attenuated inversion recovery (FLAIR) scans

T_2_-weighted images with water suppression were acquired using an axial 2D Fluid Attenuated Inversion Recovery (T_2_-FLAIR) turbo spin-echo sequence with the following scan parameters: TR = 9,000 ms, TE = 100 ms, TI = 2,499 ms, flip angle = 90°, refocusing angle = 130°, matrix size = 256 × 256, FOV = 240 × 240 mm^2^, number of slices = 32, slice thickness = 4.00 mm, BW = 287 Hz/Px, ESP = 7.17 ms, turbo factor = 16, number of averages = 1, spatial resolution = 0.94 × 0.94 × 4.00 mm^3^, TA = 5:06 min.

### High angular resolution diffusion imaging (HARDI) scans

Fifty non-collinear diffusion-encoded (b = 1,500 s/mm^2^) and five uniformly-interleaved non-diffusion-weighted (b = 0 s/mm^2^) images were acquired using an optimized q-space sampling scheme (Caruyer et al., 2013 http://www.emmanuelcaruyer.com/q-space-sampling.php) and the Human Connectome Project spin echo multi-band echo planar imaging (MB-EPI) “cmrr_mbep2d_diff” sequence (Sotiropoulos et al., 2013; Ugurbil et al., 2013) developed at the Center for Magnetic Resonance Research (Release R016 for VB17A, University of Minnesota, Minneapolis–Saint Paul, MN, USA; https://www.cmrr.umn.edu/multiband). The imaging parameters for this spin-echo MB-EPI sequence were as follows: TR = 3,284 ms, TE = 89.4 ms, flip angle = 90°, refocusing angle = 177°, matrix size = 106 × 102, FOV = 212 × 204 mm^2^, number of slices = 80, slice thickness = 2.00 mm, MB factor = 4, number of averages = 1, BW = 1,814 Hz/Px, ESP = 0.69 ms, phase partial Fourier = 6/8, spatial resolution = 2.00 × 2.00 × 2.00 mm^3^, TA = 3:17 min. However, this sequence was acquired twice, with opposite phase encoding directions—i.e., anterior-posterior (AP) and then posterior-anterior (PA)—for a total of 100 diffusion-weighted images (b = 1,500 s/mm^2^), 10 non-diffusion-weighted images (b = 0 s/mm^2^), and an overall TA = 6:34 min.

### Resting state functional MRI (rs-fMRI) scans

Resting state fMRI data were acquired using the HCP MB-EPI “cmrr_mbep2d_bold” sequence (Feinberg and Setsompop, 2013; Smith et al., 2013; Ugurbil et al., 2013) developed at the Center for Magnetic Resonance Research (Release R016 for VB17A, University of Minnesota, Minneapolis–Saint Paul, MN, USA; https://www.cmrr.umn.edu/multiband). The imaging parameters for this gradient-echo MB-EPI sequence were: TR = 1,000 ms, TE = 38.6 ms, flip angle = 61°, matrix size = 100 × 78; FOV = 220 × 171.6 mm^2^, number of slices = 80, slice thickness = 2.20 mm, MB factor = 8, number of volumes = 420, BW = 2,272 Hz/Px, ESP = 0.78 ms, EPI factor = 78, spatial resolution = 2.20 × 2.20 × 2.20 mm^3^, TA = 7:12 min.

To enable *post-hoc* EPI distortion correction of the fMRI images, two pseudo field maps with opposite phase encoding directions—i.e., left-right (LR) and right-left (RL)—were also collected with the following parameters: TR = 10,170 ms, TE = 86.6 ms, flip angle = 90°, refocusing angle = 180°, matrix size = 104 × 90, FOV = 208 × 180 mm^2^, number of slices = 72, slice thickness = 2.00 mm, MB factor = 1, number of volumes = 3, BW = 2,290 Hz/Px, ESP = 0.69 ms, EPI factor = 90, spatial resolution = 2.00 × 2.00 × 2.00 mm^3^, TA = 0.41 min each (i.e., 1:22 min to collect both).

### Pseudo-continuous arterial spin labeling (pCASL) perfusion scans

Perfusion imaging data were acquired using a MB-EPI pseudo-Continuous Arterial Spin Labeling (pCASL) sequence (Li et al., 2015a) developed at the Center for Magnetic Resonance Research (Release 1.0 for VB17A, University of Minnesota, Minneapolis–Saint Paul, MN, USA; https://www.cmrr.umn.edu/downloads/pcasl/). The scan parameters were: TR = 3,500 ms, TE = 17 ms, flip angle = 90°, matrix size = 64 × 64, FOV = 220 × 220 mm^2^, number of slices = 30, slice thickness = 4.50 mm (distance factor = 10%), MB factor = 6, number of control-tag pairs = 27, labeling duration = 1,500 ms, post-labeling delay = 1,600 ms, labeling plane offset = 88 mm from the center of the imaging slab, BW = 2,790 Hz/Px, ESP = 0.50 ms, EPI factor = 64, phase partial Fourier = 7/8, spatial resolution = 3.44 × 3.44 × 4.50 mm^3^, TA = 4:47 min.

There was a delay obtaining and installing the MB pCASL sequence on our MRI system. Therefore, because the CCOMS timeline was tied to the ongoing IMID parent study, the first 32 participants in the MS cohort were unable to receive a pCASL scan during the first of their two CCOMS MRI study visits (see Table 3).

**Table 3:** Number of participants in each cohort who passed initial quality assurance check for each scan type.

**Image type**	**Multiple sclerosis cohort**	**Healthy control cohort**	**Psychiatric cohort**
T_1_w	111	100	52
T_2_-FLAIR	111	100	52
PD/T_2_w	110	98	52
HARDI	104	100	52
rs-fMRI	109	100	50
pCASL	76	98	51
MWI	108	N/A	N/A
R${}_{2}^{*}$/QSM	48	N/A	N/A

These are the numbers of MRI scans included in the baseline CCOMS dataset, but numbers could be reduced if additional problems or artifacts are noticed during more rigorous quality checks in future analyses.

### Myelin water imaging (MWI) scans

For each participant in the MS cohort, myelin water imaging (MWI) was performed using a 3D multi-echo gradient and spin echo (mGRASE) sequence (Prasloski et al., 2012a) that has been adapted and cross-validated for Siemens 3T MRI systems (Lee et al., 2018) with the nominal following parameters: TR = 1,500 ms, number of echoes = 32, first echo time = 10 ms, ESP = 10 ms, last echo time = 320 ms, flip angle = 90°, refocusing angle = 180°, matrix size = 128 × 96, FOV = 224 × 168, number of slices = 22, slice thickness = 5.00 mm, BW = 1,260 Hz/Px, EPI factor = 3, slice partial Fourier = 6/8, spatial resolution = 1.75 × 1.75 × 5.00 mm^3^, TA = 16:53 min. Due to specific absorption rate (SAR) limits for MRI radio-frequency energy deposition (whole body < 4 W/kg and Head < 3.2 W/kg in Canada), the TR (and therefore overall scan time) for this sequence needed to be increased for some participants.

### Multi-echo gradient echo (MEGE) imaging scans

When possible in the MS cohort (i.e., remaining MRI time and participant willingness), 3D multi-echo gradient echo (MEGE) imaging was performed to facilitate QSM and R${}_{2}^{*}$ mapping. Magnitude and phase images were collected using monopolar frequency-encoding gradients without flow compensation, and the following parameters: TR = 53 ms, number of echoes = 5, TE1 = 6 ms, ESP = 10 ms, TE5 = 46 ms, flip angle = 10°, matrix size = 192 × 144, FOV = 256 × 192 mm^2^, number of slices = 96, slice thickness = 1.33 mm, BW = 190 Hz/Px, spatial resolution = 1.33 × 1.33 × 1.33 mm^3^, TA = 9:34 min.

## MRI data analysis

Image analyses were performed on Macintosh workstations running macOS 10.11 “El Capitan” or newer operating systems or Hewlett-Packard workstations running the Windows 10 Enterprise operating system, and using a combination of software packages, including: MATLAB (versions R2016a and newer; *The MathWorks Inc., Natick, MA, USA*), SPM12 (versions 6906 and newer; Wellcome Centre for Human Neuroimaging, University College London, London, UK; http://www.fil.ion.ucl.ac.uk/spm/software/spm12/), and FSL (versions 5.0 and newer; Wellcome Centre for Integrative Neuroimaging, University of Oxford, Oxford, UK; https://fsl.fmrib.ox.ac.uk/fsl/fslwiki/).

### DICOM to NIFTI file conversion

T_1_w, T_2_w, PD, T_2_-FLAIR, rs-fMRI, and pCASL images were initially converted from DICOM to NIFTI format (Li et al., 2016) using either *MRIcroGL*-embedded or standalone versions of *dcm2niix* tool (McCausland Center for Brain Imaging, University of South Carolina, Columbia, SC, USA; https://github.com/rordenlab/dcm2niix/releases), while HARDI, MWI, and MEGE images were converted using the *dicm2nii* MATLAB function (Center for Cognitive and Behavioral Brain Imaging, Ohio State University, Columbus, OH, USA; https://github.com/xiangruili/dicm2nii). After each scan, a brief quality assurance check was performed to confirm that all image files were correctly converted, that all of the expected volumes were reconstructed for the 4D images, and to identify any participants with severe artifacts (e.g., obvious participant motion, excessive signal dropout or gross geometric distortions from dental implants).

### Bias-correction, cropping, and skull-stripping of T_1_w, T_2_w, PD, and T_2_-FLAIR images (prior to lesion and tissue segmentation)

As reported previously (Marrie et al., 2021a), all T_1_w, T_2_w, PD, and T_2_-FLAIR images were initially warped to the MNI152_T1_1mm template using FSL's Linear Image Registration Tool (FLIRT; https://fsl.fmrib.ox.ac.uk/fsl/fslwiki/FLIRT) and then “defaced” to remove physically identifying features (e.g., nose, cheekbones, mouth, jaw, ears) by applying a generic image mask. After visually inspecting for accuracy, the defaced T_1_w images were cropped to remove excess neck tissue below the cerebellum by applying the bounding-box of the MNI152_T1_1mm template. The defaced and neck-cropped T_1_w images were then bias-corrected (inhomogeneity-corrected) by registering the MNI152_T1_1mm_brain to the T_1_w image [first with FLIRT, followed by FSL's Nonlinear Image Registration Tool (FNIRT; https://fsl.fmrib.ox.ac.uk/fsl/fslwiki/FNIRT)], using FSL's Automated Segmentation Tool (FAST; https://fsl.fmrib.ox.ac.uk/fsl/fslwiki/FAST) to estimate the bias-field, and then using *fslmaths* (https://fsl.fmrib.ox.ac.uk/fsl/fslwiki/Fslutils) to apply it. The defaced and neck-cropped T_1_w images were then skull-stripped using the *recon-all* function in FreeSurfer (https://surfer.nmr.mgh.harvard.edu/) to produce a brain mask for each participant that could be used to remove non-brain tissue from other imaging modalities following co-registration to the corresponding T_1_w image (Ségonne et al., 2004). The T_1_w whole-head and T_1_w brain images were ACPC-aligned and centered in MNI152 template space using a rigid-body linear registration (with 6 degrees of freedom; DOF) to the MNI152_T1_1mm template space and spline-interpolated to 1.00 mm^3^ isotropic resolution (FLIRT).

The T_2_w, PD and T_2_-FLAIR images for each participant were co-registered to the neck-cropped, bias-corrected, ACPC-aligned T_1_w image using a 6-DOF rigid-body linear registration and resampled to 1 mm isotropic resolution with spline-interpolation (FLIRT), skull-stripped by multiplying the co-registered T_1_w brain mask (*fslmaths*), and then bias-corrected (FAST).

### Lesion segmentation and filling

For participants in all three cohorts, T_2_-hyperintense lesions were initially segmented using the neck-cropped, bias-corrected, ACPC-aligned T_1_-MPRAGE and T_2_-FLAIR images as inputs to the Lesion Segmentation Tool (LST; http://www.applied-statistics.de/lst.html) in SPM12. This was achieved using the lesion growth algorithm (LGA), which first segments the T_1_w image into gray matter (GM), white matter (WM), and cerebrospinal fluid (CSF) tissue masks, calculates rough lesion probability maps based on signal intensities in the corresponding FLAIR images, thresholds these maps to create an initial (binary) lesion map (kappa initially set to 0.2 to reliably detect small lesions compared to visual inspection), and then refines these initial maps using a region-growing approach to determine the boundaries of the FLAIR hyperintensities (Schmidt et al., 2012).

Lesion masks were also created using FSL's automated Brain Intensity AbNormality Classification Algorithm (BIANCA; https://fsl.fmrib.ox.ac.uk/fsl/fslwiki/BIANCA), which is a supervised tool for white matter detection using a k-nearest neighbor algorithm (Griffanti et al., 2016). To train BIANCA, lesions were manually traced for a sub-sample of nine MS participants who had particularly large numbers of WM lesions. BIANCA lesion masks were then thresholded at 0.9999 and binarized before coregistering them (using FSL FLIRT) to each participant's ACPC space. Final lesion maps for each participant were then created as a binary cluster overlap of both the LST and BIANCA lesion maps to effectively eliminate spurious small clusters unique to only one lesion-detection technique, thereby reducing false-positives (FSL: *cluster, fslmaths*).

The final participant-specific WM masks and lesion maps were used to perform lesion filling on each participant's T_1_w image, which has been shown to improve spatial normalization, as well as GM and WM volume estimates among persons with MS (Popescu et al., 2014). This was achieved by using the lesion filling command in FSL (Battaglini et al., 2012).

### Automatic tissue classification and ROI segmentations

Automatic tissue classification — producing GM, WM, and CSF masks — was completed using FSL's FAST (Zhang et al., 2001) based on the lesion-filled T_1_w brain images and masks were created with the partial volume maps. Subcortical segmentation was performed using FSL's Integrated Registration and Segmentation Tool (FIRST; https://fsl.fmrib.ox.ac.uk/fsl/fslwiki/FIRST), a model-based segmentation tool using manually labeled anatomical segmentations that are fitted to each participant's anatomy using deformable meshes (Patenaude et al., 2011). Each participant's lesion mask was also input as control points in the registration to avoid unintentional over-weighting in lesion locations.

### Cortical thickness and volumetric analysis

Cortical thickness was measured using the *recon-all* function in FreeSurfer (http://surfer.nmr.mgh.harvard.edu/) while volumetric estimates (whole brain, gray matter, and white matter volumes) were performed using FSL SIENAX (https://fsl.fmrib.ox.ac.uk/fsl/fslwiki/SIENA) Separate volumes of the basal ganglia structures (caudate, putamen, globus pallidus, and thalamus) were obtained using FSL FIRST (https://fsl.fmrib.ox.ac.uk/fsl/fslwiki/FIRST). In addition to each participant's raw volumetric estimates, normalized volumetric estimates were also calculated using volume scaling within FSL SIENAX, which multiplies each of the raw volumes for a given participant by the scalar value of the ratio between the subject-space skull and brain volume and the MNI152_T1_2mm template skull and brain volume (Smith et al., 2001, 2002).

### Creation of calibrated T_1_w/T_2_w ratio maps

Voxel-wise T_1_w/T_2_w maps were calculated for all participants using T_1_w MPRAGE and T_2_w turbo spin-echo (TSE) images. This was achieved by first co-registering the T_2_w TSE with the T_1_w MPRAGE and then bias correcting and calibrating all of the images using the publicly-available MRTool toolbox for SPM12 (Ganzetti et al., 2014). Bias correction was performed on the T_1_w and T_2_w images using the intensity inhomogeneity correction tool in SPM12, with the bias smoothing and bias regularization parameters set at their default values (i.e., 60 mm and × 10^−4^, respectively). The bias-corrected T_1_w and T_2_w images were then intensity scaled relative to the magnitude of external landmarks using the default nonlinear histogram matching approach (i.e., based on CSF, bones, and soft tissues) to standardize their intensities before calculating the voxel-wise T_1_w/ T_2_w ratio.

### Creation of myelin water fraction (MWF), intra/extracellular water fraction (IEWF) and geometric mean T_2_ (gT_2_) maps

Voxel-wise MWI maps were computed using custom MATLAB scripts to apply a regularized non-negative least squares algorithm with stimulated echo correction, as previously reported (Prasloski et al., 2012b). Thus, MWF and IEWF were calculated as the ratio of the T_2_ distribution between 15 and 40 ms (i.e., myelin water) or 40–200 ms (i.e., intra/extracellular water) divided by the total T_2_-distribution (i.e., 15–2,000 ms). The geometric mean T_2_ for each voxel was calculated by arithmetic mean in log-space formula of the total T_2_-distribution.

### Creation of quantitative susceptibility mapping (QSM) and transverse relaxometry (R${}_{2}^{*}$) maps

QSM and R${}_{2}^{*}$ maps were generated from the MEGE images where available. QSM maps were computed using the Quantitative Susceptibility Mapping Artifact Reduction Technique (QSMART; https://github.com/MBCIU/QSMART), using the magnitude and phase MEGE images as inputs (Yaghmaie et al., 2021). First, the binary brain volume mask and brain extraction were performed using the FSL Brain Extraction Tool (BET) (Smith, 2002) and a vascular mask was created using a Frangi filter (Frangi et al., 1998). Second, an indentation mask around the cortical surfaces was generated using a Gaussian curvature method (Meyer et al., 2003; Dastan, 2020) to reduce background field removal artifacts followed by integrated Laplacian-based phase unwrapping (Li et al., 2011, 2014). Third, the background field was removed using spatially dependent filtering (Ng et al., 2011). The final QSM image was created by an offset adjustment (Sun and Wilman, 2014) of two parallel inversion steps (one with vasculature removed) done by an iterative least-squares regression (iLSQR) method to minimize streaking artifacts (Li et al., 2015b).

Voxel-wise R${}_{2}^{*}$ maps were computed using in-house MATLAB software to perform a mono-exponential fitting of the magnitude MEGE images after sinc-correction of intra-voxel linear susceptibility-induced magnetic fields (Du et al., 2009).

### Spatial normalization of T_1_w, T_2_w, PD, T_2_-FLAIR, T_1_w/T_2_w ratio, MWF, IEWF, gT_2_, QSM, and R${}_{2}^{*}$ maps

First, each participant's T_2_w, PD, T_2_-FLAIR, T_1_w/T_2_w ratio, and MWF, IEWF, gT_2_, QSM, and R${}_{2}^{*}$ images were co-registered to the corresponding lesion-filled T_1_-weighted image based on the conventional mutual information approach with default parameters in SPM12. Either the lesion-filled MPRAGE images (for participants with MS) or the raw MPRAGE images (for control participants) were then spatially normalized to the MNI template using the Computational Anatomy Toolbox for SPM12 (CAT12 version r1318; http://www.neuro.uni-jena.de/cat/). Briefly, the CAT12 Toolbox uses a combination of advanced denoising approaches (Rajapakse et al., 1997; Manjón et al., 2010), and then implements an initial affine registration (to linearly transform each participant's image to the template), followed by a two-stage non-linear normalization approach—based on Diffeomorphic Anatomical Registration Through Exponentiated Lie algebra (DARTEL) and Geodesic Shooting algorithms (Ashburner, 2007; Ashburner and Friston, 2011)—to accurately deform cortical and subcortical brain structures. As previously reported, this combination of lesion-filling and CAT12 spatial normalization yielded more consistent inter-participant agreement and lesion scaling compared to other tested spatial normalization algorithms/pipelines for participants with MS (Pirzada et al., 2020).

The CAT12 deformation field based on each participant's T_1_w image was then applied to their coregistered T_2_w, PD, T_2_-FLAIR, T_1_w/T_2_w ratio images, and (if applicable) MWF, IEWF, gT_2_, QSM, and R${}_{2}^{*}$ maps.

### HARDI data processing

All diffusion-weighted data were preprocessed using the Artifact Correction in Diffusion MRI (ACID) Toolbox in SPM12 (version beta 02; http://www.diffusiontools.com). This included simultaneous motion and eddy current correction (i.e., for each of the 110 volumes) based on whole-brain affine registrations (Mohammadi et al., 2010), and EPI distortion correction based on the oppositely phase-encoded (blip-up/blip-down) diffusion images using the Hyperelastic Susceptibility artifact Correction (HySCo) algorithm (Ruthotto et al., 2012, 2013). Whole-brain fractional anisotropy (FA), mean diffusivity (MD), axial diffusivity (AD), and radial diffusivity (RD) maps were then generated using the Fit Diffusion Tensor module and the robust weighted least-squares fitting algorithm to automatically down-weight potential remaining outliers in the diffusion signal (e.g., due to physiological noise, etc.) (Mohammadi et al., 2013). Finally, these maps were spatially normalized to the MNI template by first co-registering them to the T_1_w MPRAGE image and then applying the overall deformation field previously generated using the CAT12 Toolbox (as described Spatial normalization of T_1_w, T_2_w, PD, T_2_-FLAIR, T_1_w/T_2_w ratio, MWF, IEWF, gT_2_, QSM, and R${}_{2}^{*}$ maps).

### rs-fMRI data processing

All rs-fMRI data were initially spatially preprocessed using SPM12 (Version 7771), and then smoothed, temporally preprocessed and analyzed using the CONN Toolbox (Whitfield-Gabrieli and Nieto-Castanon, 2012) for functional connectivity analysis (Version 18b; Massachusetts Institute of Technology, Cambridge, MA, USA; http://www.nitrc.org/projects/conn). Spatial preprocessing of the rs-fMRI data included removing the first 10 s (i.e., first 10 brain volumes) to eliminate volumes that may not have reached a magnetic steady-state, motion correction (by realigning each volume to the mean T${}_{2}^{*}$-weighted image), EPI distortion correction based on the oppositely phase-encoded (blip-up/blip-down) and the Hyperelastic Susceptibility artifact Correction (HySCo) algorithm (Ruthotto et al., 2012, 2013), coregistration of the T_1_w MPRAGE image (and the forward deformation field previously generated using the CAT12 Toolbox, as described above in Spatial normalization of T_1_w, T_2_w, PD, T_2_-FLAIR, T_1_w/T_2_w Ratio, MWF, IEWF, gT_2_, QSM, and R${}_{2}^{*}$ Maps to the mean T${}_{2}^{*}$-weighted image, and spatial normalization of the coregistered MPRAGE and all of the motion- and HySCo-corrected fMRI volumes to the MNI template (by applying the coregistered forward CAT12 deformation). The rs-fMRI images were then smoothed with a 4.0 mm full-width half-maximum (FWHM) 3D Gaussian kernel.

The spatially preprocessed rs-fMRI images were then brought into the CONN toolbox, where the Artifact Detection Tool (http://www.nitrc.org/projects/artifact_detect/, Massachusetts Institute of Technology, Cambridge, MA*)* was used to identify and regress out specific time points with scan-to-scan intensity changes of z > 3.0, translational motion > 0.50 mm in any direction, and/or rotational motion >0.05 degrees in any plane. However, given that numerous functional connectivity studies have highlighted the importance of correcting for even small amounts of participant motion (Power et al., 2012; Satterthwaite et al., 2012; Van Dijk et al., 2012) and physiological noise (Shmueli et al., 2007; Chang and Glover, 2009), additional temporal preprocessing was performed to regress out the effects of participant motion (i.e., the time-courses of all 6 realignment parameters) and physiological noise [i.e., the time-courses of eroded white matter and cerebrospinal fluid masks using the aCompCor method (Behzadi et al., 2007)]. Finally, the rs-fMRI data were temporally band-pass filtered (0.008–0.09 Hz) to isolate low frequency fluctuations of interest (Biswal et al., 1995).

### pCASL data processing

All pCASL data was initially split into two 4D images (i.e., a pCASL image from volumes 1–53, and M_0_ reference image from volumes 54–55). Both images were spatially cropped to remove the five most inferior slices, which were contaminated with signal from labeling RF modulation. The pCASL volumes were motion-corrected using FSL's MCFLIRT (https://fsl.fmrib.ox.ac.uk/fsl/fslwiki/MCFLIRT). The M_0_ reference image was linearly coregistered (6-DOF) to the anatomical T_1_w image with the boundary-based registration (BBR) option using FSL's FLIRT. This registration was subsequently applied to the cerebral blood flow images.

The pCASL data were subsequently processed using the ASLtbx2 toolbox (Wang et al., 2008; Wang, 2012) in SPM12 (https://cfn.upenn.edu/zewang/ASLtbx.php). Motion correction (*spm_realign_asl* tool) was performed after removing apparent motion caused by signal fluctuations related to the control-tag pattern from the estimated motion parameters. The images were realigned to the mean image using a two-pass procedure. High-pass temporal filtering was performed with a first order Butterworth filter with a cutoff frequency of 0.0057 Hz (*ASLtbx_asltemporalfiltering* tool). The data were spatially smoothed with a 5 mm FWHM Gaussian kernel. Surround subtraction was then used to generate a perfusion-weighted time series. Cerebral blood flow (in units of ml/100 g tissue/min) was then estimated, assuming a label efficiency of 0.85 and approximating the longitudinal magnetization M_0_ from the control images (*asl_perf_subtract* tool, edited to allow for multi-band slice timing).

## Future statistical analyses and sample sizes

Future reports will describe the cognitive, psychiatric, and vascular risk characteristics of the study cohorts using means (standard deviations), median (interquartile range) and frequency (percent). Bivariate analyses will use ANOVA, Kruskal-Wallis tests, chi-square tests, Fisher's exact tests and correlations as appropriate. We will examine the cross-sectional and longitudinal associations between vascular and psychiatric comorbidity and imaging measures using multivariate regression approaches including multivariate analysis of covariance, canonical correlation analysis and quantile regression analysis. We will conduct mediation analyses to determine if the effects of psychiatric and vascular comorbidity on cognition are mediated by imaging measures. Potential covariates in these analyses will include age, gender, cohort, and medication use.

Table 3 suggests that the current dataset will be suitably powered to detect various differences and correlations with small-to-medium effect sizes (Cohen, 1988). For example, using the “pwr” package in R (R: A Language and Environment for Statistical Computing; https://www.R-project.org), the minimum sample sizes for one-tailed and two-tailed tests using one-sample (paired) *t*-tests, two-sample (unpaired) *t*-tests, and correlations with a statistical power of 0.8 and a significance level of 0.05 are as follows:

- One-Sample *T*-Test with a small effect size (Cohen's *d* ≥ 0.3): Minimum sample size is *N* < 70 at both time points for one-tailed tests, and *N* < 89 at both time points for two-tailed tests.
- Two-Sample *T*-Test with a medium effect size (Cohen's *d* ≥ 0.5): Minimum sample size is *N* < 50 per group for one-tailed tests, and *N* < 63 per group for two-tailed tests.
- Correlations with a medium effect size (*r* = 0.3): The minimum sample size is *N* < 66 for one-tailed tests, and *N* < 84 for two-tailed tests.

## Discussion

The onset, time course, and severity of MS-related cognitive impairments are heterogeneous, and physical and psychiatric comorbidities may account for clinically meaningful portions of these observed differences. However, previous studies examining the association between comorbidities and cognition in MS have been largely cross-sectional, while longitudinal studies have been limited (Ruano et al., 2017; Marrie et al., 2018a, 2021a,c). Moreover, most MS neuroimaging studies have included only persons with MS and perhaps healthy controls, often explicitly excluding participants with psychiatric conditions from these groups. Inclusion of intermediate control groups, such as persons with diagnosed psychiatric disorders or chronic health conditions other than neurological disease, allows for the contributions of comorbidities in MS to be fully explored. Therefore, the longitudinal design and subsequent inclusion of healthy and psychiatric control groups are important and unique aspects of this study.

Another important component of this study is the breadth of MRI sequences that were acquired. This will enable many different aspects of brain macrostructure, microstructure, functional connectivity, and perfusion to be investigated relative to the clinical, cognitive, and psychiatric measures obtained. Common macroscopic morphological measures include voxel-wise or regional estimates of brain volume or cortical thickness (Ashburner and Friston, 2000; Fischl and Dale, 2000; Whitwell, 2009), as well as estimates of more global volumetric measures (e.g., deep gray matter, cortical gray matter, normal appearing white matter, and/or lesion volumes). The myriad of quantitative imaging measures will also facilitate local estimates of microstructural characteristics such as tissue density, iron concentration, and myelin density. Indeed, the microstructural MRI sequences included in this protocol were chosen because they have different sensitivity vs. specificity tradeoffs, and are known to probe different microstructural characteristics. Some of these metrics, such as T1w/T2w ratio and the diffusion tensor imaging measures, are thought to be sensitive to disease-related microstructural alterations but lack pathophysiological specificity (Uddin et al., 2018, 2019), while other metrics, such as myelin water fraction and quantitative susceptibility, may have lower sensitivity but are able to provide specific information about local myelin and iron concentrations, respectively (Laule et al., 2006; Langkammer et al., 2012). In addition, given the prevalence of perivascular inflammation and perivenular lesions in MS, the availability of perfusion MRI data will allow for examination of relatively under-studied topics such as potential differences, or longitudinal changes, in cerebral perfusion in relation to specific cognitive and psychiatric measures (Lapointe et al., 2018). The resting-state fMRI data permit a wide variety of analysis methods including seed-based, network-based, independent component analysis-based, and graph-based approaches to characterize alterations in brain connectivity (Lee et al., 2013). With such a variety of complimentary MRI measures available to shed light on various aspects of brain structure and function, along with the fact that there are longitudinal data from the MS cohort and cross-sectional data from both healthy and psychiatric control groups, future CCOMS studies will seek to investigate specific neural correlates of MS-related psychiatric comorbidities and cognitive changes.

The CCOMS study design and methodology are consistent with addressing several of the “key priorities” for understanding cognitive deficits in MS previously outlined by Sumowski and colleagues (Sumowski et al., 2018), including:

1. ***Aiming to distinguish between “neuroimaging correlates” and the “neural*** ***bases” of cognitive deficits:*** The current study addressed this by including certain imaging methods (e.g., myelin water imaging, arterial spin labeling, etc.), which can be used—in combination with the longitudinal design—to ascribe specific anatomical and physiological underpinnings (i.e., regional myelin concentration and blood perfusion, respectively) to cognitive decline.
2. ***Investigation of “isolated cognitive constructs (e.g., memory)”, rather than*** **“*heterogeneous composites of multiple cognitive domains”:*** The cognitive tests included in the current study were selected to assess different cognitive domains commonly affected by MS (Sumowski et al., 2018), including memory, processing speed, and executive function; and the psychiatric evaluations were conducted to assess severity of depression and anxiety symptoms in addition to presence or absence of disorders.
3. **“*Development of multivariate models*** ***(incorporating demographic, neuroimaging and clinical variables) to better*** ***predict decline”****:* The demographic and clinical histories obtained from each participant, the availability of multiple structural and functional MRI measures, as well as the longitudinal neuroimaging component and extensive psychiatric and cognitive evaluations will enable multivariable analyses; and the inclusion of comorbid conditions in these models will also play a central role.
4. ***The development and use of “clinically feasible tools for the quantification*** ***of disease burden from MRIs”:*** All of the neuroimaging methods used in the current study were implemented using a clinical 3T MRI system with conventional hardware (described in the “MRI data acquisition” section). Therefore, similar neuroimaging protocols (including the advanced MRI sequences) should be feasible at most major hospitals and MRI research centers.
5. ***Addressing data reproducibility issues by “promoting (or at least enabling)*** ***multicenter collaborations with comparable methods”:*** By presenting the CCOMS MRI data acquisition, and image processing pipelines in greater detail than is typically possible in manuscripts presenting study-specific findings, we hope to facilitate future studies with similar study designs and methods—which would enable direct comparisons and meta-analyses.

Despite careful study planning, obtaining a wide variety of structural and functional MRI data, and using advanced works-in-progress (WIP) and customer-to-customer (C2P) MRI pulse sequences whenever possible, there are limitations of this study. First, because the control groups were added later, only when additional funding became available, longitudinal data collection from these groups was not possible, and there were also some small but statistically significant differences in the proportion of female participants, age, and education level between the MS and HC cohorts. Second, due to the reasonably long scan time, complete imaging datasets were not acquired for all participants (Table 3). Participants without T_1_w, T_2_w-FLAIR, PD/T_2_w, HARDI or rs-fMRI data were almost exclusively due to failed *post hoc* quality assurance checks for subject motion or other artifacts. However, there were also systematic differences for the pCASL and R${}_{2}^{*}$ sequences. As noted earlier, there was a problem with the multi-band pCASL license file that could not be resolved before nearly one-third of participants in the MS cohort had already been scheduled for their first MRI study visit, as this schedule was determined by the IMID study protocol. There was also a lower number of R${}_{2}^{*}$ scans among MS participants due to time constraints. This reflected a number of factors such as participants arriving late for their study; any issues with the MRI system, the need to increase the length of the MWI scan to lower SAR among participants with elevated body mass index, or if one or more of the earlier scans needed to be repeated due to motion- or other artifacts that were noted during the scan time. Finally, there were some irregularly occurring image artifacts observed in the earliest axial T_1_w MPRAGE images. However, these artifacts would not have materially affected the image processing pipelines for the various microstructural and functional imaging methods included in the study, and we confirmed that there was no significant effect on total intracranial volume estimates (see Supplementary material), and this artifact was eliminated by switching one of the 3D phase-encoding directions to acquire sagittal images for all subsequent study participants (including all of the control participants, as well as all 2-year follow-up scans among participants in the MS cohort).

## Conclusions

The Comorbidity and Cognition in Multiple Sclerosis (CCOMS) study is a longitudinal MRI study of the association between comorbidity and cognition in MS which includes healthy and psychiatric control groups, detailed psychiatric and cognitive evaluations, and a variety of advanced macrostructural, microstructural, functional, and perfusion MRI methods. By providing a detailed description of the CCOMS MRI hardware, pulse sequences, acquisition parameters, and image processing pipelines, this protocol should serve as a reference for subsequent CCOMS papers examining MRI-based neural correlates of clinical, cognitive, and neuropsychiatric characteristics, as well as providing technical guidance for studies from other labs to facilitate reproducibility.

## Ethics statement

The studies involving human participants were reviewed and approved by University of Manitoba Health Research Ethics Board, and all participants provided their written informed consent to participate in this study.

## Author contributions

MNU, TDF, JK, ELM, CAH, CO'G, RP, LAG, JMB, JJM, CNB, JDF, RAM, and CRF contributed to the study design. All authors participated in data acquisition and/or analysis, manuscript preparation and/or editing, and approved the submitted draft of the manuscript.

## Funding

This work was supported by a Waugh Family Foundation Multiple Sclerosis Society of Canada operating grant (EGID-2639), the Waugh Family Chair in Multiple Sclerosis (to RAM), the Canadian Institutes of Health Research (THC-135234), Crohn's and Colitis Canada, Research Manitoba, the Natural Sciences and Engineering Research Council of Canada, the Health Sciences Centre Foundation, and the Brain Canada Foundation.

## Conflict of interest

CNB has consulted to Abbvie Canada, Amgen Canada, Bristol Myers Squibb Canada, Janssen Canada, Pfizer Canada, Roche Canada, Sandoz Canada, Takeda Canada, Mylan Pharmaceuticals, and Avir Pharmaceuticals. He has received unrestricted educational grants from Abbvie Canada, Janssen Canada, Pfizer Canada, Takeda Canada and Bristol Myers Squibb Canada. He has received investigator initiated grants from Abbvie Canada, Pfizer Canada, Amgen Canada, and Sandoz Canada. He has been on speaker's bureau of Abbvie Canada, Janssen Canada, Pfizer Canada, and Takeda Canada. JF receives research grant support from the Canadian Institutes of Health Research (CIHR), the National Multiple Sclerosis Society, the Multiple Sclerosis Society of Canada, Crohn's and Colitis Canada, Research Nova Scotia; consultation and distribution royalties from MAPI Research Trust. CRF receives research funding from the Brain Canada Foundation, MS Society of Canada, Natural Sciences and Engineering Research Council of Canada (NSERC), Research Manitoba, and Health Sciences Centre Foundation. He also serves on the Editorial Board of Scientific Reports (Radiology Section) and is an Associate Editor for Frontiers in Neurology (Section on Applied Neuroimaging). ELM receives research funding from NSERC and St. Francis Xavier University, and other grant funding from the Council of Atlantic University Libraries. JK receives research funding from the MS Society of Canada, University of Manitoba and Health Sciences Centre Foundation. JMB receives research funding from CIHR, Brain and Behavior Research Foundation and the MS Society of Canada. JMM has conducted trials for Biogen Idec and Roche, and receives research funding from the MS Society of Canada. LAG receives research funding from CIHR, the MS Society of Canada and the Health Sciences Centre Foundation. She has consulted to Roche Canada. MRE receives research funding from Manitoba Public Insurance and the Brain Canada Foundation. He is the Deputy Editor for the Journal of Magnetic Resonance Imaging and serves on the Editorial Boards of Investigative Radiology, Insights into Imaging, and Der Radiologe. He has also served on both the Scientific Advisory Board and Imaging Biomarkers Subcommittee of the European Society of Radiology (ESR), and is currently a member of the Research Advisory Committee of the Radiological Society of North America (RSNA). He has received speaking honoraria from Bayer Healthcare, Canon Medical Systems, Olea Medical, and Siemens Healthcare. RAM receives research funding from CIHR, Research Manitoba, Multiple Sclerosis Society of Canada, Multiple Sclerosis Scientific Foundation, Crohn's and Colitis Canada, National Multiple Sclerosis Society, The Consortium of Multiple Sclerosis Centers, The Arthritis Society, US Department of Defense and UK MS Society. She serves on the Editorial Board of Neurology and Multiple Sclerosis Journal. She is a co-investigator on studies funded in part by Biogen Idec and Roche no funds to her or her institution. RP receives research funding from the Workers Compensation Board of Manitoba. The remaining authors declare that the research was conducted in the absence of any commercial or financial relationships that could be construed as a potential conflict of interest.

## Publisher's note

All claims expressed in this article are solely those of the authors and do not necessarily represent those of their affiliated organizations, or those of the publisher, the editors and the reviewers. Any product that may be evaluated in this article, or claim that may be made by its manufacturer, is not guaranteed or endorsed by the publisher.
