# Supplementary material for: The comorbidity and cognition in multiple sclerosis (CCOMS) neuroimaging protocol: Study rationale, MRI acquisition, and minimal image processing pipelines
Source: Front Neuroimaging. 2022 Aug 24;1:970385. doi: 10.3389/fnimg.2022.970385 (PMC10406313; doi:10.3389/fnimg.2022.970385)
Supplement: Supplementary file 1 [file Data_Sheet_1.PDF]

Supplementary Material

Some irregularly occurring ringing artifacts were observed in the T_1w MPRAGE images for a subset of MS participants enrolled early in the study. This issue was not apparent in the initial pilot testing data, and was therefore not noticed until *post hoc* quality assurance checks began for the CCOMS study participants.

Upon further testing, it was determined that these artifacts resulted from a unique combination of the axial MPRAGE acquisition parameters, the 32-channel head coil (i.e., they could not be reproduced when using a standard 12-channel head coil), and participant head anatomy/orientation. We found that changing to sagittal acquisitions, using non-selective inversion recovery pulses, and implementing anterior-posterior phase encoding eliminated these artifacts. Since the MPRAGE sequence is 3D (with one frequency-encoding and two phase-encoding directions), one of the phase-encoding directions remained unchanged, while the initial frequency-encoding and the other phase-encoding directions switched when moving from axial to sagittal acquisitions. Given that signals were strongly T_1-weighted (TR = 1900 ms; TE = 2.47 ms) and the readouts were kept as short as possible (GRAPPA = 2; Gradient Mode = Fast; Echo Spacing = 7.3 ms), all images should be fairly insensitive to magnetic susceptibility distortions; and because all other acquisition parameters remained constant, image signal-to-noise ratio and tissue contrast are expected to be similar for the axial and sagittal acquisitions.

Nonetheless, because of the relatively low image bandwidth (170 Hz/Px), it is possible that changing one of the MPRAGE phase-encoding directions could produce slightly different B_0-related distortions between the axial and sagittal acquisitions (Van

der Kouwe et al., 2008). We therefore acquired both axial and sagittal T_1w MPRAGE data from 32 Healthy Control and Psychiatric Control study participants during their study visits and compared the resulting images. Even when the axial image artifacts were evident in this sub-sample, no artifacts were observed in the sagittal MPRAGE images (**Supplementary Figure 1A**), so the decision was made to acquire only the sagittal images for the remainder of the study.

To compare whether the different T_1w acquisition schemes had any systematic effects on brain volumetric estimates, we computed the volumes of the resulting brain tissue segmentations, as described previously in Section 5.4 for all 32 participants' axial and sagittal T_1w images. We then computed the relative differences (i.e., $[\text{Volume}_{\text{Axial}} - \text{Volume}_{\text{Sagittal}}] \div [(\text{Volume}_{\text{Axial}} + \text{Volume}_{\text{Sagittal}}) \div 2]$) between paired estimates of total intracranial volume (gray matter + white matter + cerebrospinal fluid volume), brain parenchymal volume (gray matter + white matter volume), and cerebrospinal fluid volume across participants [mean ± SD] (**Supplementary Figure 1B**).

Although there were no significant differences in estimated total intracranial volumes (mean relative difference = 0.02 ± 0.43%; two-tailed paired t-test $p = 0.70$), there were small but significant differences in the estimated total parenchymal volumes (mean relative difference = 1.00 ± 0.99%; two-tailed paired t-test $p < 0.01$) and estimated total cerebrospinal fluid volumes (mean relative difference = −3.47 ± 2.60%; two-tailed paired t-test $p < 0.01$). Due to the inherent confound between potential differences in readout-dependent B_0-distortions versus the artifacts themselves, it is difficult to definitively determine the extent to which each of these may have driven the small but statistically significant differences in tissue classifications. However, the image artifacts (when

observed) most commonly ran along and through gray matter/cerebrospinal fluid and gray matter/white matter interfaces (red arrows in **Supplementary Figure 1A**), with the artifact often appearing as regions of decreased signal (dark bands) within the brain parenchyma. Based on this, the most likely cause of the slightly higher cerebrospinal fluid estimates (and correspondingly lower parenchymal volume estimates) in the axial MPRAGE images are artifact-related tissue misclassifications, where brain parenchyma with decreased signal intensity was erroneously classified as cerebrospinal fluid. Moreover, the fact that the sagittal vs. axial image cerebrospinal fluid volume estimates appeared to have similar lower-bound and median values, but the axial images resulted in a larger fourth quartile range and high-value outliers (**Supplementary Figure 1B**), is consistent with our initial subjective assessment that artifacts were only observed in a subset of the axial images. Taken together, we therefore suggest that the majority of the estimated differences – on average 1.00% for brain parenchymal volume and -3.47% for cerebrospinal fluid volume – are most likely driven by the subset of axial MPRAGE images with the most prominent artifacts.

Finally, having conducted this comparison, we note that most of the T_1w images – including those for all control participants and all of the 2-year follow-up scans among participants in the MS cohort – were acquired with the artifact-free sagittal acquisition scheme. Put another way, all study participants have artifact-free cross-sectional T_1w imaging data, and many of the participants in the MS cohort have artifact-free axial and/or sagittal T_1w imaging data as well.

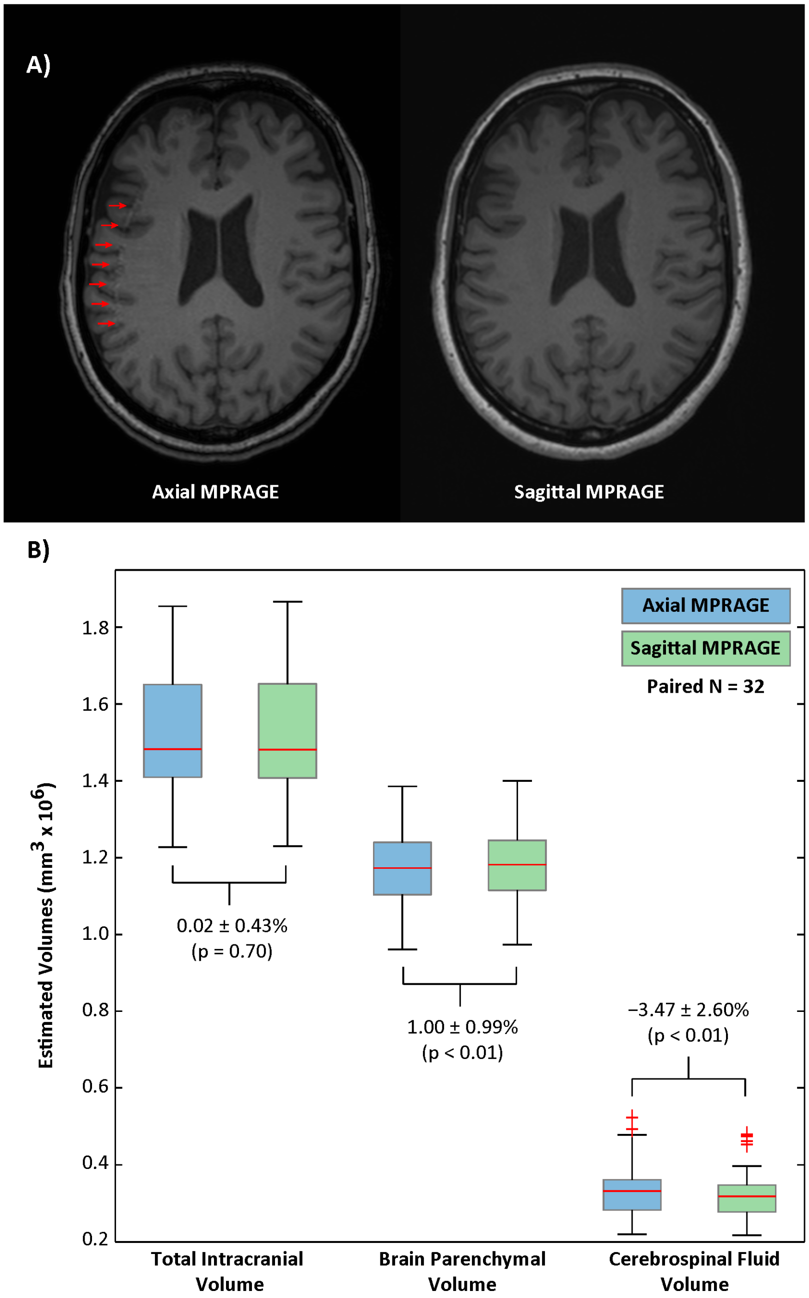

Supplementary Figure 1: A) Prominent example of the Axial MPRAGE image artifact that was observed in a sub-set of early study participants, and B) Comparison of Axial vs. Sagittal MPRAGE segmented tissue volumes from 32 control participants for whom data were collected both ways. Note: differences are reported as a percentage [mean ± SD], and p-values are based on two-tailed, paired-sample t-tests.

Supplementary Reference

Van der Kouwe, A.J., Benner, T., Salat, D.H., Fischl, B., 2008. Brain morphometry with multiecho MPRAGE. Neuroimage 40, 559-569.
